# Pre- and Postnatal Nutritional Histories Influence Reproductive Maturation and Ovarian Function in the Rat

**DOI:** 10.1371/journal.pone.0006744

**Published:** 2009-08-25

**Authors:** Deborah M. Sloboda, Graham J. Howie, Anthony Pleasants, Peter D. Gluckman, Mark H. Vickers

**Affiliations:** 1 The Liggins Institute and the National Research Centre for Growth and Development, The University of Auckland, Auckland, New Zealand; 2 AgResearch, Hamilton, New Zealand; Institute of Clinical Effectiveness and Health Policy, Argentina

## Abstract

**Background:**

While prepubertal nutritional influences appear to play a role in sexual maturation, there is a need to clarify the potential contributions of maternal and childhood influences in setting the tempo of reproductive maturation. In the present study we employed an established model of nutritional programming to evaluate the relative influences of prenatal and postnatal nutrition on growth and ovarian function in female offspring.

**Methods:**

Pregnant Wistar rats were fed either a calorie-restricted diet, a high fat diet, or a control diet during pregnancy and/or lactation. Offspring then were fed either a control or a high fat diet from the time of weaning to adulthood. Pubertal age was monitored and blood samples collected in adulthood for endocrine analyses.

**Results:**

We report that in the female rat, pubertal timing and subsequent ovarian function is influenced by the animal's nutritional status *in utero*, with both maternal caloric restriction and maternal high fat nutrition resulting in early pubertal onset. Depending on the offspring's nutritional history during the prenatal and lactational periods, subsequent nutrition and body weight gain did not further influence offspring reproductive tempo, which was dominated by the effect of prenatal nutrition. Whereas maternal calorie restriction leads to early pubertal onset, it also leads to a reduction in adult progesterone levels later in life. In contrast, we found that maternal high fat feeding which also induces early maturation in offspring was associated with elevated progesterone concentrations.

**Conclusions:**

These observations are suggestive of two distinct developmental pathways leading to the acceleration of pubertal timing but with different consequences for ovarian function. We suggest different adaptive explanations for these pathways and for their relationship to altered metabolic homeostasis.

## Introduction

The past century has seen a dramatic decline in the age of menarche: in Europe the age of menarche has fallen from 17 to ∼12.5 years of age [1]–[5], although the rate of decline is slowing [6], [7]. This fall is usually attributed to improvements in child health and nutrition since the early 19^th^ century, when the age of menarche was highest. This led to the hypothesis that the age of menarche is directly linked to a critical degree of body fat [8]; however although prepubertal nutrition and the age of puberty are certainly associated in some way, this concept did not stand up to critical analysis of the data [9].

Data in humans suggest the possibility that complex interactions between prenatal and postnatal events influence the timing of puberty within one generation [2], [10], [11]. In the 1948 UK birth cohort, both being born smaller and accelerated weight gain in childhood led to independent and interactive effects on the age of menarche [12], and we have recently confirmed this observation in an Australian cohort [13]. Opposing influences of prenatal and postnatal growth have previously been described for adrenarche [14], central fat distribution [15] and insulin sensitivity at age 8 years [16]. In data derived from adoption studies [17], [18], young girls who migrated from underprivileged countries to developed countries entered puberty significantly earlier than girls who remained in their country of origin [19], [20], and in unrelated studies such children have shown persistent differences in ovarian function later in life [11].

It has been argued that the effects of intrauterine cues such as prenatal undernutrition on later metabolic function have an adaptive origin [21], in that they are a result of evolved processes designed to maintain reproductive fitness across a range of potential environments. Gluckman *et al*. [22] have proposed that the fetus uses nutritional signals (and/or changes in the endocrine environment) to anticipate its future energetic environment, and through developmental plasticity adjusts its phenotype accordingly [23], [24]. In this regard, developmental plasticity would confer a phenotype that is better suited for the environment and thereby increase the organism's chances of reproducing successfully. Life history theory links earlier ages of maturation to ecological circumstances including energy availability, risks of predation and other causes of extrinsic mortality [25]. Part of an adaptive response to early life nutritional challenge might therefore be expected to be an acceleration in the age of reproductive maturation, provided that the organism can metabolically sustain earlier reproduction without further compromising its own viability.

In general, life history concepts suggest that poor nutrition or threatening circumstances in early life lead to accelerated maturation, through which the organism trades body size and longevity for earlier reproduction in a threatening environment [26]. There are a wealth of data showing that in the rat, compromising early life signals such as maternal undernutrition or glucocorticoid exposure lead to offspring that develop a deleterious metabolic phenotype; including obesity, with both peripheral components such as sarcopenia and insulin resistance, and central components including hyperphagia, a preference for fatty foods and altered willingness to exercise [27]–[29]. More recently, the effects of relative ‘overnutrition’ have come into question. Of current concern in developed countries are the rising rates of obesity [30] and gestational diabetes, both of which produce offspring who develop obesity [31]–[35]. While it has been suggested that body fat and therefore childhood nutrition *per se* play a direct role in determining the age of pubertal onset [36], [37], the effects of an intrauterine obesogenic environment have not been considered.

The overall aim of the present study was to investigate the impact of differing nutritional exposures (standard rat chow, high fat or caloric restriction) during intrauterine and early postnatal life on pubertal onset and ovarian function in female rat offspring. Additionally, we investigated whether a lifetime of high fat consumption by the dam has differing effects compared to high fat consumption that is restricted to pregnancy and lactation. After birth, offspring were further challenged by a diet that was either standard rat chow or high in fat. We evaluated growth, timing of reproductive maturation and adult ovarian function. We report that early life caloric restriction, either before birth or during lactation, consistently accelerates the age of maturation. Although we report that the combination of prenatal caloric restriction and postnatal high fat nutrition also advanced the age at puberty, this was not directly linked to body weight at puberty. Intriguingly, maternal high fat consumption also accelerated reproductive maturation in female offspring, but here a post-weaning high fat diet had limited additional effect. We also present data indicating that these two early life nutritional exposures had differential effects on later life ovarian function, suggestive of two developmental pathways that affect the tempo of reproductive maturation in the rat.

## Results

### Altered maternal nutrition modifies offspring growth

**Figure 1:**
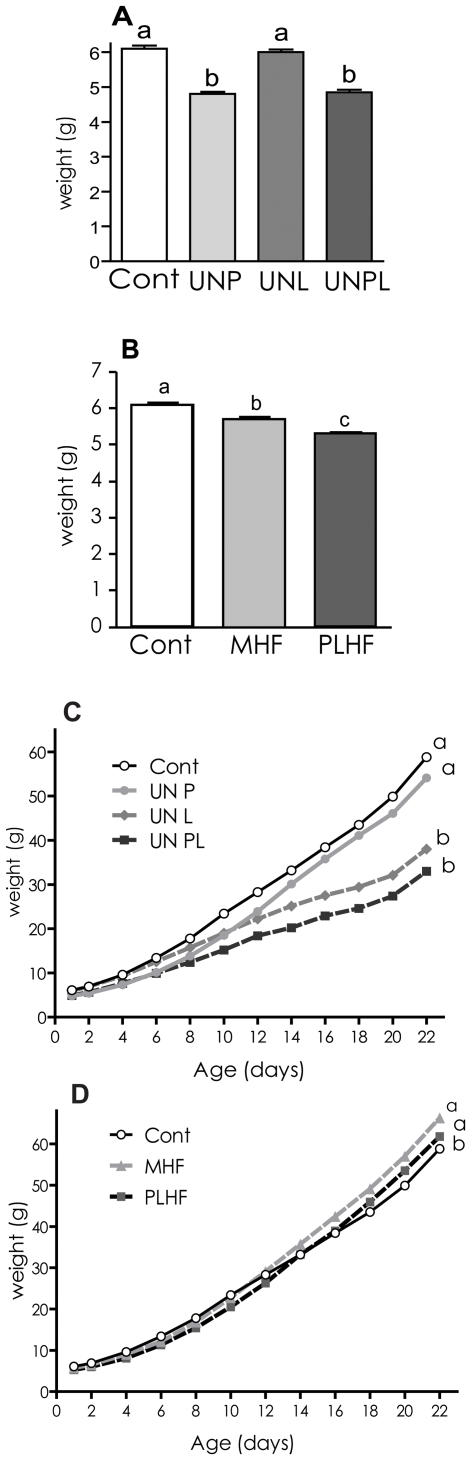
Offspring birth weights and neonatal growth curves in all groups. A) Birthweight in offspring of dams that were undernourished during pregnancy and/or lactation; B) Birthweight in offspring of dams that were fed a high fat diet; C) Neonatal growth curves in offspring of undernourished dams; D) Neonatal growth curves in offspring of high fat fed dams. Cont – control pregnancies; UN P – dams undernourished during pregnancy only; UN L – dams undernourished during lactation only; UN PL – dams undernourished during pregnancy and lactation; MHF – dams fed a high fat diet pre-conceptionally and throughout pregnancy and lactation; PLHF – dams fed a chow diet pre-conceptionally and a high fat diet throughout pregnancy and lactation only. Data are expressed as group means±SEM. Groups denoted by different letters are significantly different at p<0.05.

Birth weights of offspring born to rat dams that were undernourished during pregnancy were significantly reduced compared to those of control pregnancies (p<0.05, Figure 1A). Between birth and postnatal day 22, pups born to dams that were calorie restricted during pregnancy and/or lactation remained lighter than controls (Figure 1C), and were significantly lighter at weaning (day 22) compared to ad-libitum fed controls (Cont 59.1±0.7 g, UNP 54.1±0.8, UNL 38.5±0.9 g, UNPL 33.0±0.7; p<0.05). Comparatively, maternal high fat nutrition led to a small but significant (p<0.05) reduction in birth weight compared to Cont (Figure 1B). There was a further significant (p<0.05) reduction in birth weight of female offspring born to PLHF dams compared to MHF and Cont offspring. In contrast to calorie restricted offspring, these pups regained their weight and by day 12 (MHF) or day 14 (PLHF) surpassed controls in both weight and rate of weight gain, reflective of a significantly altered growth trajectory (Figure 1D). At weaning, HF offspring weights were significantly increased compared to controls (Cont 59.1±0.7 g, MHF 66.3±0.9 g, PLHF 63.8±0.7 g, p<0.0001 and p<0.05 versus controls respectively).

### Pubertal onset and adult ovarian function is dependent on maternal nutritional background

#### Pubertal onset in control offspring

Pubertal age of offspring born to control dams was similar to that reported previously [38]. A post-weaning HF diet alone advanced the age of puberty in female offspring born to dams fed a control diet during pregnancy and lactation (p<0.001, Table 1).

**Table 1:** Age and weight at puberty in female offspring.

Groups	Pubertal Age (days)	Pubertal Weight (g)
**Cont+C**	34.6±0.5	121.5±3.7
**Cont+HF**	32.8±0.4	116.7±6.3
**UN P+C**	33.3±0.3	111.6±5.6
**UN P+HF**	31.8±0.2	105.9±2.4
**UN L+C**	33.5±0.3	94.6±3.8
**UN L+HF**	31.8±0.3	92.1±2.8
**UN PL+C**	33.3±0.4	84.9±3.6
**UN PL+HF**	30.7±0.3	77.9±3.3
**MHF+C**	33.0±0.3	114.4±2.4
**MHF+HF**	32.6±0.7	122.5±5.0
**PLHF+C**	32.7±0.4	108.6±2.7
**PLHF+HF**	31.4±0.5	103.8±3.8

effect of maternal diet across groups, p<0.001.

effect of postnatal diet in undernourished and control animals, p<0.001.

effect of postnatal diet in undernourished and control animals, NS.

#### Pubertal onset in nutritionally challenged offspring

**Figure 2:**
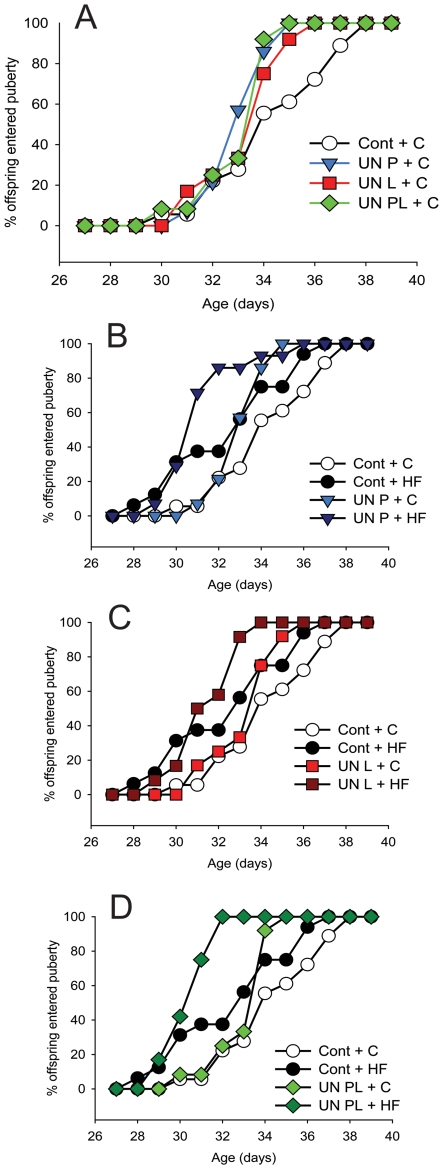
Percentage of offspring entering puberty over time in animals whose dams received 50% of normal nutrition. (A) female offspring of all groups fed a chow diet; (B, C, D) female offspring fed a postnatal high-fat diet. Cont – control pregnancies; UN P – dams undernourished during pregnancy only; UN L – dams undernourished during lactation only; UN PL – dams undernourished during pregnancy and lactation. C-postnatal chow diet; HF – postnatal high fat diet. Data represent at least six litters per maternal dietary group. Maternal diet effect; p<0.001; postnatal diet effect; p<0.001.

Irrespective of postnatal diet, changes in early life nutrition resulted in the early onset of puberty. Maternal nutrient restriction during pregnancy and/or lactation significantly advanced pubertal age in female offspring to a similar degree (p<0.01, figure 2A; Table 1). In calorie restricted offspring (UNP, UNL, UNPL), a post-weaning HF diet further advanced the age at puberty (p<0.001; Figure 2B, C, D; Table 1) compared to those offspring who were normally nourished *in utero* and fed a HF diet post-weaning (Table 1).

**Figure 3:**
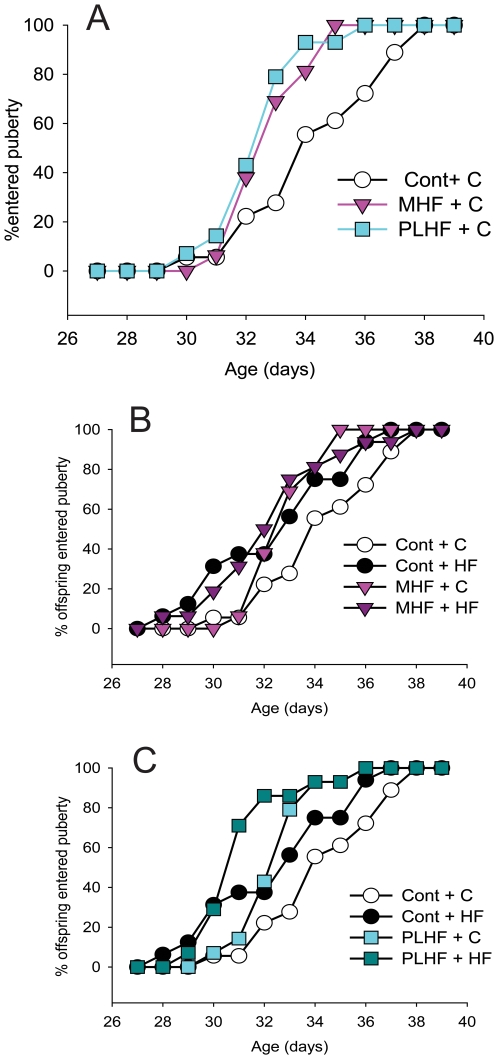
Percentage of offspring entering puberty over time in animals whose dams received a high fat diet. (A) female offspring in all groups fed a chow diet; (B, C, D) female offspring fed a postnatal high-fat diet. Cont – control pregnancies; MHF – dams fed a HF diet pre-conceptionally and throughout pregnancy and lactation; PLHF – dams fed a chow diet pre-conceptionally and a HF diet through pregnancy and lactation only. C – postnatal chow diet; HF – postnatal high fat diet. Data represent at least six litters per maternal dietary group. Maternal diet effect p<0.001, postnatal diet effect p<0.001 in control offspring - in maternal high fat offspring postnatal diet had no effect. *Note that Control offspring in Figure 3 and Figure 4 are the same animals.

Maternal consumption of a HF diet both prior to and during pregnancy and lactation, or only during pregnancy and lactation, significantly advanced the age of puberty in female offspring (p<0.001; Figure 3A; Table 1). A post-weaning HF diet in offspring exposed to a prenatal HF diet had no further effect (Figure 3B, C; Table 1).

**Figure 4:**
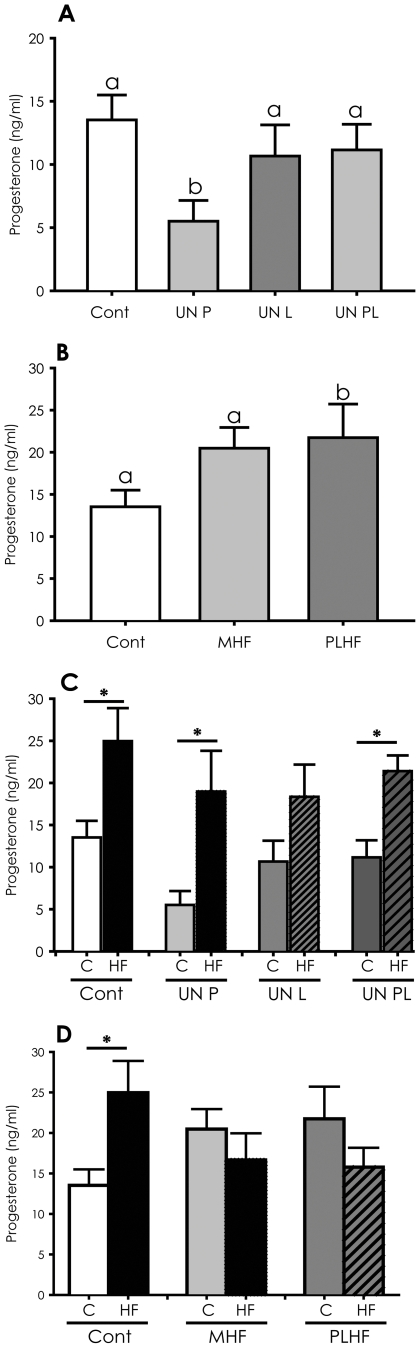
Circulating adult female progesterone concentrations at proestrous. (A) female offspring whose dams received 50% of normal nutrition and who were fed either a postnatal standard chow diet or (C) a high fat postnatal diet, and (B) offspring of dams fed a high fat diet who were fed a postnatal standard chow diet or (D) a high fat postnatal diet. Cont – control pregnancies; UN P – dams undernourished during pregnancy only; UN L – dams undernourished during lactation only; UN PL – dams undernourished during pregnancy and lactation. C-postnatal chow diet; HF – postnatal high fat diet. MHF – dams fed a high fat diet pre-conceptionally and throughout pregnancy and lactation; PLHF – dams fed a chow diet pre-conceptionally and a high fat diet through pregnancy and lactation only. Data are expressed as group means±SEM. Groups denoted by different letters are significantly different at p<0.05. * denotes p<0.05. *Note that Control offspring in Figure 3 and Figure 4 are the same animals.

Circulating progesterone concentrations in adult female offspring born to dams that were undernourished during pregnancy (UNP) were significantly lower than concentrations in Cont, UNL or UNPL offspring (p<0.05, Figure 4A). A post-weaning HF diet significantly increased progesterone concentrations in Cont and UN animals (p<0.05, Figure 4C), although in UNL offspring these differences did not reach statistical significance. Maternal consumption of a HF diet resulted in adult offspring progesterone concentrations that were significantly higher than those of offspring born to Cont dams (p<0.05; Figure 4B), although in MHF offspring these differences did not reach statistical significance. A post-weaning HF diet in offspring exposed to a prenatal HF diet had no further effect (Figure 4D).

### Weight at puberty

Offspring that were exposed to altered maternal nutrition (either maternal undernutrition or high fat diet) all showed significantly lower body weights at pubertal onset compared to Cont offspring (p<0.05; Table 1).

## Discussion

These data demonstrate that intrauterine, lactational and post-weaning nutritional histories make differential contributions to the age of puberty in the female rat, and lead to persistent effects on ovarian function extending into adulthood. While a post-weaning high fat diet had the effect of accelerating puberty, this was restricted to only those offspring of control dams or those born to dams that were calorie restricted, and in calorie restricted offspring this effect was relatively small compared to that of early life nutrition.

### Maternal undernutrition

Fetal growth restriction as a consequence of impaired intrauterine conditions can be interpreted as part of a life history strategy in which the organism anticipates a shorter life because of a higher risk of extrinsic mortality, and therefore invests less into growth but accelerates maturation to ensure reproductive fitness [10], [36], [39]–[41]. Consistent with this, we observed that both prenatal and lactational undernutrition accelerated puberty – although only the former was accompanied by the later development of obesity and other components of metabolic compromise, as previously reported [29]. The shift towards an earlier puberty may represent a life course strategy [10] intended to preserve fitness [36], [41] by reducing the risk of death before reproducing, or by allowing a greater number of successful reproductive episodes before death. We speculate that the well reported phenomenon of metabolic programming as a result of poor prenatal nutrition might be a secondary outcome of a life history strategy to accelerate maturation. A fitness advantage would be obtained only if there were sufficient nutrient stores to support earlier reproduction, and an integrated adaptive response would thus require both accelerated puberty and altered metabolism. However, an accelerated reproductive tempo may not be maintained in the face of later nutritional constraints. There may be a postnatal override such that if energetics remain poor during postnatal life, reproductive maturation may be delayed until the nutritional environment is improved sufficiently to support a first pregnancy. Using a similar experimental paradigm, we have previously demonstrated that prenatal undernutrition in a rodent model was associated with a prepubertal increase in food intake [29] and alterations in epigenetic regulation of and mRNA levels of hepatic genes to favour lipogenesis [42]. Taken together, our observations could suggest that the prenatal undernutrition-induced acceleration in pubertal onset observed in the present study may contribute to a significant drive to alter metabolic capacity. These data therefore may be consistent with clinical observations that that lower but normal birth weight followed by accelerated weight gain up to age 8 is associated with earlier menarche [12], [13].

Mechanisms regulating pubertal onset are no doubt multifactorial. Leptin is a permissive neuro-regulatory factor for the onset of puberty [43], [44]. It synchronizes growth and fertility with periods of either adequate or inadequate food availability [45]; post-weaning treatment with leptin accelerates puberty in rodents [46], [47], and leptin in the rat neonate affects hypothalamic maturation [48]. Since in the present study neonatal growth trajectories in pups that were exposed to maternal undernutrition began to deviate from those of control pups at a time that coincides with a neonatal surge in circulating leptin concentrations, which itself is nutritionally influenced [49], [50], leptin may be a central driver in initiating maturational changes. This is consistent with our previous observations that these offspring have altered leptin regulation and are hyperleptinemic as adults [51].

In the present study, body weight did not predict the age at puberty. Indeed those offspring, for which maternal calorie restriction was extended into lactation, did not show obesity as adults, but still displayed accelerated reproductive maturation. The observation that lactational undernutrition can lead to a modification of the adult metabolic phenotype is compatible with work by others showing that postnatal undernutrition can modify metabolic programming [52], [53]. It does appear however that such modification does not reverse the altered tempo of reproductive maturation in programmed offspring. Using a life-history interpretation, adaptive developmental responses to environmental cues may be graded; according to the severity of the stress experienced [22], [54]–[56]. The differing outcomes between each of the perinatal undernutrition approaches reported here are likely to reflect these confounders of severity and timing.

### Maternal high fat exposure

We found that exposure to a high fat diet either throughout the lifetime of the mother or restricted to pregnancy and/or lactation led to early pubertal onset. These findings are compatible with a previous report of early puberty following high fat nutrition during pregnancy [57]. High fat perinatal nutrition advanced the age at puberty to the same degree as did post-weaning high fat nutrition in control offspring, and intriguingly the addition of a high fat postnatal diet following high fat prenatal nutrition had no further effect on pubertal age. Therefore, nutritionally-induced accelerated reproductive maturation following high fat exposure may not be limited to one distinct critical developmental window.

In our model, early life signals of high fat nutritional conditions led to accelerated maturation. We have previously shown that offspring of dams fed a high fat diet throughout life (including during pregnancy and lactation) show accelerated weight gain in postnatal life, independent of postnatal diet [58]. In the present study, offspring of nutritionally challenged dams develop obesity; thus a perpetuation and possibly a compounding of accelerated pubertal onset may ensue in the next generation as these offspring will themselves be fatter at an earlier reproductive age.

### Ovarian function

Maternal exposure to either caloric restriction or to high fat nutrition was associated with accelerated puberty in female offspring, but there were distinct differences in mature ovarian function as measured by progesterone concentrations in the proestrous phase. The study design did not allow for a direct measure of reproductive performance, however, high salivary progesterone concentrations in women is associated with greater ovulation rates and ovulatory events are associated with childhood and adult environmental and ecological factors [58]. Although we did not measure reproductive performance, we speculate that the higher progesterone concentrations observed may be associated with better functioning of the corpora lutea. We recognise however that the influence of central gonadal drivers cannot be discounted.

In contrast to MHF and PLHF offspring, those born to dams that were undernourished during pregnancy showed lower adult circulating progesterone concentrations. In life history terms, the trade-off for those born to dams undernourished during pregnancy may be an earlier maturation but a faster decline in ovarian function with aging, associated with significantly reduced adult progesterone levels. There are data that suggest that at least in mice, aging *per se* may be accelerated in offspring of poorly nourished dams. Ozanne *et al.* [59] have reported that prenatal undernutrition, in contrast to postnatal undernutrition, leads to reduced longevity in mice. In our study, while puberty was accelerated in maternal high fat exposed offspring, ovarian function was maintained or enhanced into adult life. We speculate that whereas pubertal onset was accelerated in the UNP group to maintain some potential for reproduction in an environment predicted to have a higher mortality risk, in the HF offspring maturation was accelerated to opportunistically enhance fitness.

Finally, while the use of rodent models for reproductive investigation is generally well accepted, a number of differences must be recognized. Rodents for instance have short reproductive cycles closely linked with circadian rhythms, and do not experience menses or true “menopause” as a result of follicular depletion as typically observed in humans, although follicular depletion does occur. Although we accept that rodents and humans may differ in their adaptive responses to early life cues, there are many reports outlining associations between a suboptimal early life environment and altered phenotype in both species [60], [61].

### Conclusions

In summary, we have demonstrated that in the rat, pre- and postnatal nutritional histories together influence both ovarian function and the tempo of reproductive maturation in female offspring. In our study, both maternal high fat nutrition and diminished maternal calorie intake impacted on female offspring reproductive maturation resulting in early pubertal onset, most likely through two different, potentially adaptive, pathways. We speculate that our findings may imply adaptive responses to early life cues predicted by a life-history approach, although confirmation of these predictions requires further studies to assess reproductive fitness. Future studies will elucidate the long term effects of maternal nutrition on offspring reproductive capability and to determine the molecular mechanisms underlying early pubertal onset.

## Materials and Methods

### Ethics Statement

All animal work was approved by the Animal Ethics Committee of the University of Auckland.

### Animals

In the present study, we used an established model of developmental programming via maternal nutrient manipulation [29], [62]. Wistar rats (age 100±5 days) were time mated using a rat estrous cycle monitor to assess the stage of estrous before introducing the male. After confirmation of mating, rats were housed individually in standard rat cages with free access to water. All rats were kept in the same room with a constant temperature maintained at 25°C and a 12-h light, 12-h dark cycle. Animals were assigned to one of 6 nutritional groups: 1) dams fed a standard diet (protein 18%, fat 5%, digestible energy 3.4 kcal/gm, *Teklad Global 18% Protein Diet, Diet 2018*) *ad libitum* throughout pregnancy and lactation (**Cont** group), 2) undernourished dams fed 50% of a standard diet throughout pregnancy and lactation (**UNPL** group), 3) undernourished dams fed 50% of a standard diet throughout pregnancy only (**UNP** group), 4) undernourished dams fed 50% of a standard diet throughout lactation only (**UNL** group), 5) dams fed a high-fat diet throughout pregnancy and lactation (**PLHF** group; 45% kcal as fat, 20% protein; 4.73 kcal/gm, *Research Diets Inc. D12451*) and 6) dams fed a pre-conceptional high-fat diet, from the time of their weaning (22 days of age) through to conception and throughout pregnancy and lactation (**MHF** group; 45% kcal as fat, 20% protein; *Research Diets Inc. D12451*). Food intake was recorded daily until the end of pregnancy. After birth, pups were weighed and litter size adjusted to eight pups per litter (4 male and 4 female) to ensure standardised nutrition until weaning at day 22. At weaning, all offspring were weight matched within maternal dietary groups and placed on either standard rat chow or a high-fat diet (**HF**; *Research Diets Inc. D12451*, 45% kcal as fat), resulting in a total of 12 groups (Table 2). From postnatal day 27, offspring were checked daily for markers of reproductive maturity: vaginal opening and canalisation in females and balanopreputial separation in males. At 150 days of postnatal age animals were DEXA scanned for body composition, then fasted overnight and killed by injection of pentobarbitone (60 mg/kg, s.c.) anaesthesia followed by decapitation. Blood was collected into heparinised Vacutainer tubes and stored on ice until centrifugation and removal of plasma for analysis.

**Table 2:** Description of early life nutrition and offspring groups.

Pre-pregnancy	Pregnancy	Lactation	Offspring Postnatal Diet	Group (sample size)
100% Control Diet			Control	Cont (18)
			High Fat	Cont+HF (16)
100% Control Diet	50% Control Diet	100% Control Diet	Control	UNP (14)
			High Fat	UNP+HF (14)
100% Control Diet		50% Control Diet	Control	UNL (12)
			High Fat	UNL+HF (12)
100% Control Diet	50% Control Diet		Control	UNPL (12)
			High Fat	UNPL+HF (12)
100% Control Diet	100% High Fat Diet		Control	PLHF (16)
			High Fat	PLHF+HF (16)
100% High Fat Diet			Control	MHF (16)
			High Fat	MHF+HF (16)

#### Estrous Staging in Adult Offspring

In order to assess stage of estrous cycle in adult offspring, vaginal smears were collected at the time of post mortem after pentobarbitone injection prior to decapitation. Briefly, each animal was laid in a supine position and samples were obtained by inserting the tip of a 100 ul pipette tip approximately 2–4 mm into the vagina, releasing sterile water (∼50 ul) and immediately drawing back to collect the sample. Vaginal smears were initially evaluated under wet conditions where the sample was placed on a glass microscope slide and examined under a light microscope at 20–40×magnification. After determination of estrous stage, samples were air dried and stained using an adaptation of the Papanicolaou (PAP) stain used for humans [63]. Smears were then visualised under a light microscope and digital images captured, which were re-assessed for the proportion of three cell types: epithelial cells, cornified cells, and leukocytes. Determination of estrous stage was assessed as previously described [63], [64].

#### Progesterone Measures

Circulating progesterone concentrations were measured in adult female offspring in the proestrous stage. Measures were made using mass spectrometry. The internal standard (IS) was corticosterone-d8. Briefly, 100 µL of IS (20 ng/mL in water) was added to 200 µL plasma or standard mixture in a glass tube. Steroids were immediately extracted into ethyl acetate (1 mL). The supernatant was removed to a new tube then dried in a centrifugal vacuum drier. The contents were resuspended in 100 µL of mobile phase (80% methanol and 20% water) and transferred into high performance liquid chromatography (HPLC) injector vials. 30 µL aliquots were injected into an HPLC mass spectrometer system consisting of a Waters Alliance 2690 Separations Module (Waters Corporation, Milford, MA, USA), followed by an Ion Max APCI source on a Finnigan TSQ Quantum Ultra AM triple quadrapole mass spectrometer, all controlled by Finnigan Xcalibur software (Thermo Electron Corporation, San Jose, CA, USA). The mobile phase flow rate was 600 µL/min through a Luna 3 µ C18(2) 100A 250×4.6 mm column held at 40°C (Phenomenex, Auckland, New Zealand). Retention times (Rt) were: 7.0 minutes for corticosterone-d8 and 12.5 minutes for progesterone. Ionization was in positive mode and Q2 had 1.2 mTorr of argon. The mass transitions followed were: 315.1 → 109.0 at 28V for progesterone and 355.3 → 125.2 at 24V for corticosterone-d8. The intra- and inter-assay coefficients of variation were 4.2%, and 9% respectively.

### Statistical Analyses

Statistical analyses were carried out using StatView (Version 5, SAS Institute, Cary, NC) statistical packages. All data were tested for normality prior to analyses. Differences between groups were determined by ANOVA with maternal background, gender and diet as factors; where appropriate differences were observed at p<0.05 level, post-hoc analyses were performed (Fishers PLSD (Protected Least Significant Difference)). Pubertal data are represented as group means and as percentage of offspring entering puberty as a proportion of total. Statistical significance was accepted at the p<0.05 level.
